# Effectiveness of Pulsed Electromagnetic Field Therapy on Neuropathic Pain: A Systematic Review and Meta-Analysis

**DOI:** 10.3390/neurolint18020028

**Published:** 2026-02-06

**Authors:** Jesus Antonio Lara-Reyes, Cristofer Zarate-Calderon, Gonzalo E. Aranda-Abreu, Luis I. García, Fausto Rojas-Durán

**Affiliations:** Instituto de Investigaciones Cerebrales, Universidad Veracruzana, Xalapa 91190, Mexico; jlara@uv.mx (J.A.L.-R.); garanda@uv.mx (G.E.A.-A.);

**Keywords:** neuropathic pain, pulsed electromagnetic field, PEMF, spinal pain, diabetic neuropathy, neuromodulation, non-invasive therapy

## Abstract

Background: Neuropathic pain represents a substantial global burden with limited effective therapeutic options. Pulsed Electromagnetic Field (PEMF) therapy has emerged as a potential non-invasive adjuvant, though clinical evidence remains inconsistent. This systematic review and meta-analysis evaluated PEMF efficacy and safety, specifically analyzing the influence of etiology and stimulation parameters. Methods: Following PRISMA 2020 guidelines (PROSPERO: CRD420251184151), five databases (Cochrane, PubMed, Scopus, Web of Science, and LILACS) were searched for Randomized Controlled Trials (RCTs) comparing PEMF versus sham. Risk of bias was assessed via Cochrane RoB 2, and heterogeneity was explored through detailed subgroup analyses. Results: Thirteen RCTs met the inclusion criteria (*N* = 688). While global analysis indicated a statistically significant pain reduction (SMD: −1.01; *p* = 0.03), it exhibited extreme statistical heterogeneity (I^2^ = 92.8%) and instability. After adjusting for missing studies using the Trim-and-Fill method, global significance disappeared. However, subgroup analysis resolved this inconsistency, revealing a massive, clinically meaningful effect in Spinal/Radicular pain (SMD: −2.35; 95% CI: −4.42 to −0.29), whereas Peripheral Neuropathy showed no significant reduction (SMD: −0.38; 95% CI: −0.86 to 0.10). Conclusions: The PEMF evidence base for neuropathic pain is currently highly fragmented. Extreme heterogeneity and publication bias render “one-size-fits-all” efficacy estimates invalid and potentially misleading. Instead, our data reveals a critical etiological divergence: PEMF appears highly effective for spinal/radicular pathology, likely due to the mechanical nature of the lesion, but demonstrates limited efficacy for diffuse peripheral neuropathy. Future research must abandon generic protocols in favor of etiology-specific trials, prioritizing high-frequency parameters and rigorous bias control.

## 1. Introduction

Neuropathic pain is a chronic and debilitating condition arising from a lesion or disease of the somatosensory nervous system. Clinically, this disorder is characterized by the presence of positive symptoms such as allodynia, hyperalgesia, and dysesthesia, which imposes a profound burden on patients’ quality of life [1,2].

Globally, it represents a considerable challenge for public health. Although prevalence estimates vary widely depending on the methodology (ranging from 0.9% to 17.9%), more precise analyses suggest a prevalence of 6–8%, a figure expected to escalate due to population aging and the rising incidence of diabetes [3,4]. Notably, recent Global Burden of Disease data highlighted that nervous system conditions, including neuropathic pain, were the leading global cause of years lived with disability in 2021, showing an 18.2% increase since 1990 [5,6].

The clinical urgency of this problem lies in the fact that neuropathic pain is often more intense, refractory, and difficult to treat than non-neuropathic pain [1,2]. Current therapeutic options, including first-line drugs such as tricyclic antidepressants, α2δ ligands, and serotonin-norepinephrine reuptake inhibitors, often yield suboptimal outcomes and present undesirable side effects that limit their long-term clinical utility [7,8]. This situation underscores a substantial unmet clinical need for effective and safe treatments [8].

Consequently, research into non-pharmacological interventions has gained critical relevance. Pulsed Electromagnetic Field (PEMF) therapy is a non-invasive neuromodulation modality that has garnered attention for its potential to alleviate pain. Mechanistically, it is postulated that PEMF acts through multifaceted pathways, encompassing influence on microcirculation, reduction in inflammation, promotion of tissue repair, and modulation of neuronal excitability [9,10,11,12,13,14,15]. Specifically, evidence suggests that PEMF exposure can influence peripheral nerves and sensory neurons through the modification of membrane potential, suggesting a direct action in pain transmission [15].

Despite these plausible mechanisms, clinical evidence regarding its efficacy has been inconsistent. Some works have reported encouraging results, suggesting benefits especially in short-term applications or conditions such as chronic low back pain with a neuropathic component [14,16]. However, the literature also highlights important inconsistencies, as several studies indicate that PEMF application does not always confer a statistically significant benefit compared to sham (placebo) [17]. From a regulatory perspective, these discrepancies are reflected in the current status of the technology; while the U.S. Food and Drug Administration (FDA) and European regulatory bodies have cleared specific PEMF devices for bone healing and post-operative pain or edema [18], their dedicated indication for neuropathic pain remains largely off-label or jurisdiction-dependent. This regulatory gap further emphasizes the necessity of a systematic synthesis to clarify the real clinical reach of the therapy.

This variability in results is largely attributable to critical methodological differences. Previous studies have commingled distinct neuropathic pain etiologies and used widely diverse stimulation parameters (frequency, intensity, duration), making it difficult to establish clear conclusions [14,19,20]. Furthermore, the technical challenge of administering a truly indistinguishable sham (placebo) is a recognized limitation in electromagnetic therapy trials; sensory or auditory cues can often compromise participant blinding, potentially inflating the placebo response [16,21]. Furthermore, the certainty of the available evidence is often low according to the GRADE system, preventing the formulation of definitive clinical recommendations [21].

To resolve these discrepancies, it is essential to perform an analysis that not only synthesizes the data but systematically explores the causes of variability. In this context, the present work aims to determine the efficacy and safety of PEMF therapy in reducing neuropathic pain compared to sham stimulation. Considering clinical and technical particularities, the study provides a stratified approach that differentiates between etiology and frequency parameters. Aligned with PRISMA 2020 guidelines and the GRADE framework, it seeks to elucidate whether the treatment offers a real clinical benefit, simultaneously examining its impact on quality of life and its safety profile.

## 2. Materials and Methods

### 2.1. Study Design and Registration

This systematic review and meta-analysis were conducted in strict adherence to the Preferred Reporting Items for Systematic Reviews and Meta-Analyses (PRISMA) 2020 guidelines [22]. The completed PRISMA 2020 checklist is provided as Supplementary Material File S1. The study protocol was prospectively registered in the International Prospective Register of Systematic Reviews (PROSPERO) under the identifier CRD420251184151.

### 2.2. Search Strategy and Information Sources

A comprehensive literature search was executed across five major bibliographic databases: Cochrane Central Register of Controlled Trials (CENTRAL), PubMed, Scopus, Web of Science (SCI), and LILACS. To mitigate publication bias and ensure literature exhaustiveness, gray literature and ongoing trial registries were queried via ClinicalTrials.gov and Google Scholar. The search timeframe encompassed all literature published from 1 January 2000, through 10 November 2025, with no language restrictions imposed. The search strategy was developed based on the PICO framework, combining Medical Subject Headings (MeSH) and free-text terms related to “pulsed electromagnetic field”, “neuropathic pain”, and “neuropathy”. The complete search strings for each database are provided in Supplementary Material File S2.

### 2.3. Inclusion and Exclusion Criteria

Studies were selected based on the following inclusion criteria:

- Study Design: Randomized controlled trials (RCTs), including both parallel-group and crossover designs.
- Population: Adult participants (≥18 years) with confirmed clinical diagnosis of chronic neuropathic pain (defined as pain persisting for ≥3 months) of either peripheral or central etiology.
- Intervention: Non-invasive Pulsed Electromagnetic Field (PEMF) therapy, regardless of specific stimulation parameters (frequency, intensity, or waveform).
- Comparators: For the primary efficacy analysis, the control group was required to receive sham (placebo) stimulation. For the assessment of adjuvant effectiveness, studies comparing PEMF plus Treatment as Usual (TAU) versus TAU alone were eligible.
- Outcomes: Studies reporting quantitative pain intensity data measured by validated scales.

Exclusion criteria encompassed studies involving pediatric populations, animal models, or healthy volunteers. Trials focusing exclusively on acute pain (<3 months) or mixed pain populations (e.g., non-specific low back pain) were excluded unless data for a specific neuropathic subgroup could be explicitly extracted. Furthermore, to isolate the electromagnetic effects, studies comparing PEMF against other active stimulation modalities without a sham control were excluded.

### 2.4. Study Selection and Data Extraction

Following the removal of duplicate records using Mendeley Reference Manager (v2.139.0), two independent reviewers screened titles and abstracts against the eligibility criteria. Potentially eligible studies were retrieved for full-text assessment. Any discrepancies regarding study eligibility were resolved through consensus or consultation with a third senior reviewer.

Data extraction was performed independently by two reviewers using a standardized, pre-piloted form. Extracted domains included: (1) Study Characteristics (author, year, country, design); (2) Population (sample size, age, sex, and specific etiology of neuropathic pain to facilitate subgroup stratification); (3) Intervention Parameters (frequency [Hz], magnetic flux density [Tesla/Gauss], waveform, session duration, and treatment regimen); and (4) Outcomes (mean and standard deviation [SD] for pain intensity change, and adverse events). For studies presenting data exclusively in graphical format, numerical values were extracted using WebPlotDigitizer software (Version 5.2) [23].

### 2.5. Quality and Certainty of Evidence Assessment

The methodological quality of the included RCTs was independently assessed by two reviewers using the Cochrane Risk of Bias tool (RoB 2) [24]. Bias was evaluated across five domains: randomization process, deviations from intended interventions, missing outcome data, measurement of the outcome, and selection of the reported result. The overall certainty of evidence for the primary outcome (pain intensity reduction) was evaluated using the Grading of Recommendations Assessment, Development and Evaluation (GRADE) framework [25], classifying evidence as high, moderate, low, or very low based on risk of bias, inconsistency, indirectness, imprecision, and publication bias.

### 2.6. Statistical Analysis

All statistical analyses were conducted using R software (Version 4.4.3) within the RStudio environment (Version 2025.09.2+418), utilizing the meta and metafor packages. Given the anticipated clinical heterogeneity across neuropathic conditions and stimulation protocols, a random-effects model was applied for all meta-analyses to provide a conservative estimate of the treatment effect. For continuous outcomes, the treatment effect was expressed as the Standardized Mean Difference (SMD) with 95% confidence intervals (CI), applying Hedges’ g correction to adjust for small sample sizes. For dichotomous outcomes, the Risk Ratio (RR) was calculated.

To ensure directional consistency, scales where higher scores indicated better health were inverted (multiplied by −1) so that negative effect sizes consistently represented symptom improvement. Standard Errors (SEM) were converted to Standard Deviations (SD) where necessary. Statistical heterogeneity was quantified using the Chi^2^ test and the I^2^ statistic, with values >50% indicating substantial heterogeneity.

To address sources of variance, pre-specified subgroup analyses were stratified by: (1) Clinical Etiology (Peripheral Neuropathy vs. Spinal/Central Pain); and (2) Intervention Parameters (Low-Frequency vs. High-Frequency/Radiofrequency). Imputation of missing standard deviations was strictly avoided if the proportion of missing data exceeded 30% of the included studies; in such instances, quantitative synthesis was omitted in favor of narrative synthesis to preserve statistical integrity. Publication bias was assessed using contour-enhanced funnel plots and Egger’s regression test. Upon detection of asymmetry, a Trim-and-Fill sensitivity analysis was conducted to adjust for potential missing studies.

## 3. Results

### 3.1. Search Results and Study Selection

**Figure 1:**
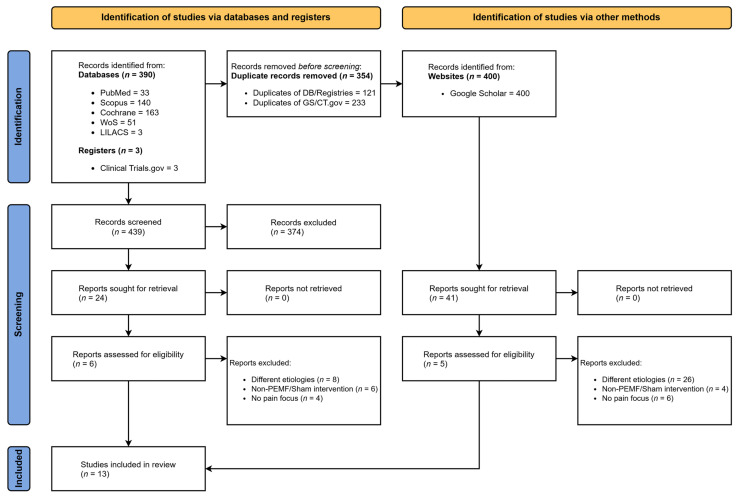
PRISMA 2020 flow diagram. The schematic illustrates the study selection process, detailing the number of records identified, screened, and included in the systematic review and meta-analysis.

The systematic literature search initially identified 1056 records from bibliographic databases. Supplementary searches of gray literature and trial registries yielded an additional 6434 records. Following the removal of duplicates and the application of automated filters, 354 unique citations were screened by title and abstract. Of these, 65 articles were deemed potentially relevant and retrieved for full-text assessment. Fifty-three studies were subsequently excluded, primarily due to ineligible study designs, mixed pain populations lacking subgroup data, or the absence of a sham comparator. Ultimately, 13 randomized controlled trials (RCTs) met all inclusion criteria [10,26,27,28,29,30,31,32,33,34,35,36,37]. Nine of these studies (*N* = 688) provided sufficient quantitative data to be included in the primary meta-analysis of efficacy [10,26,27,29,31,34,35,36,37]. The study selection process is illustrated in Figure 1.

### 3.2. Characteristics of Included Studies

The pooled population for the quantitative synthesis comprised 688 adult participants with a mean age ranging from 34.2 to 65.8 years, and a sex distribution of approximately 55% female. The included trials reflected the heterogeneous nature of neuropathic pain conditions. The most prevalent etiology was diabetic peripheral neuropathy (k = 5), followed by spinal/radicular pain (k = 3), and carpal tunnel syndrome (k = 1). Intervention protocols exhibited significant technical variability. Stimulation frequencies ranged from the extremely low-frequency (ELF) domain (<100 Hz) to radiofrequency (27.12 MHz). Treatment intensity also varied, with session durations spanning 10 to 120 min and total intervention periods ranging from 10 days to 18 weeks. Regarding comparators, all nine studies in the primary efficacy analysis utilized sham devices to ensure participant blinding, while studies assessing adjuvant effectiveness (k = 4) compared PEMF combined with usual care against usual care alone.

### 3.3. Risk of Bias Assessment

**Figure 2:**
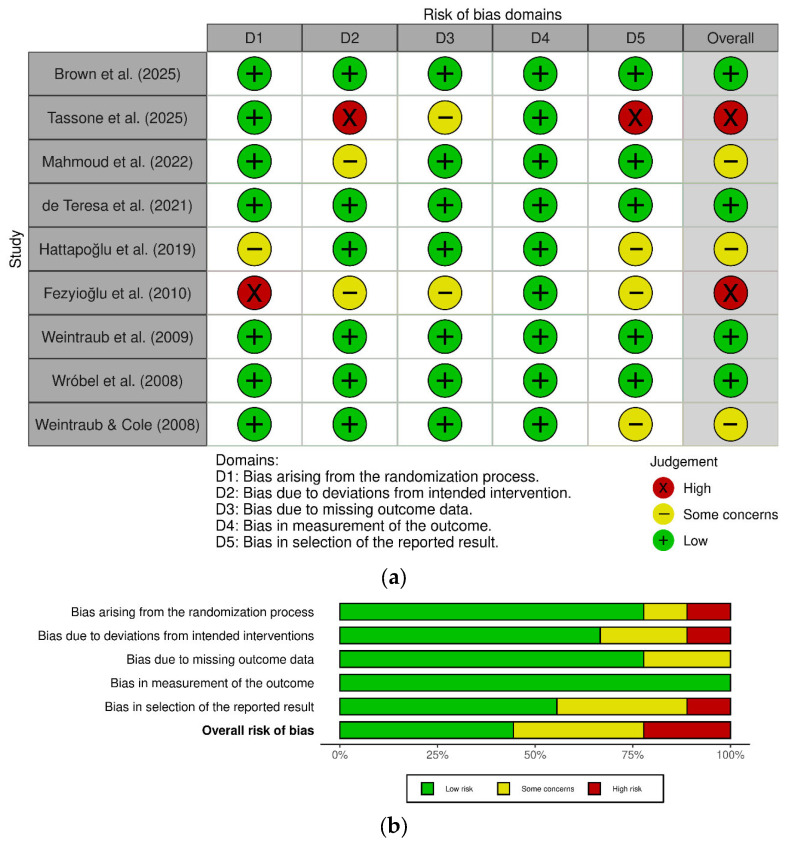
Risk of bias assessment. (**a**) Risk of bias graph: review authors’ judgements about each risk of bias item presented as percentages across all included studies [10,26,27,29,31,34,35,36,37]. (**b**) Risk of bias summary: review authors’ judgements about each risk of bias item for each included study (assessed using the Cochrane RoB 2 tool [Version 22 August 2019]).

The methodological quality of the nine RCTs included in the quantitative synthesis is summarized in Figure 2. The risk of bias profile was variable: four studies (44%) were classified as having a low overall risk of bias, three studies (33%) raised some concerns, and two studies (22%) were deemed high risk. The “some concerns” classification was largely driven by insufficient reporting of the randomization process (Domain 1). Conversely, the measurement of the outcome (Domain 4) was robust across all trials (100% low risk), reflecting the consistent use of validated patient-reported outcome measures and effective blinding of assessors.

### 3.4. Primary Efficacy Analysis: PEMF vs. Sham

In the primary meta-analysis (k = 9; *N* = 688), PEMF therapy was associated with a statistically significant reduction in pain intensity compared to sham stimulation (SMD: −1.01; 95% CI: −1.90 to −0.11; *p* = 0.03). However, this estimate was characterized by extreme statistical heterogeneity (I^2^ = 92.8%; *p* < 0.001).

**Figure 3:**
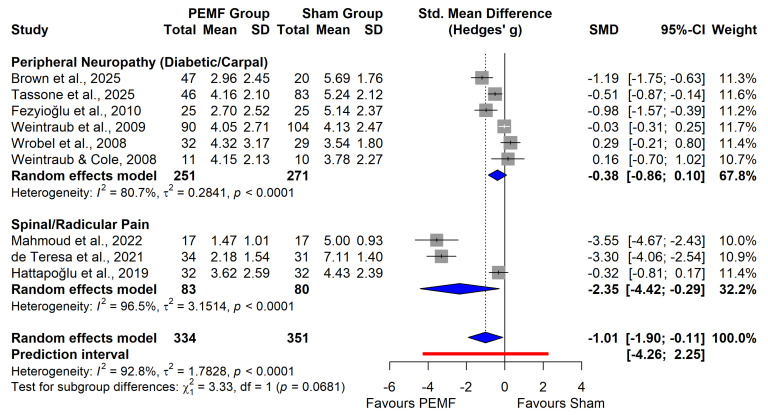
Plot of pain intensity by etiology. Meta-analysis of the effect of PEMF versus Sham on pain intensity, stratified by neuropathic pain etiology (Peripheral Neuropathy vs. Spinal/Radicular Pain) [10,26,27,29,31,34,35,36,37].

Furthermore, the prediction interval spanned from −4.26 to 2.25, crossing the line of no effect (Figure 3). This wide interval is a critical finding, implying that the expected efficacy of PEMF in an unselected, generic neuropathic pain population is highly unpredictable and could range from strongly therapeutic to negligible or even null. This statistical uncertainty underscores the necessity of the subsequent subgroup analysis to identify specific responders and reinforces that the global average should not be used for individual clinical prognosis without considering the underlying etiology.

#### 3.4.1. Subgroup Analysis by Etiology

Stratification by pathophysiology revealed a clinically important divergence in treatment response (Figure 3). The subgroup of patients with Spinal/Radicular Pain (k = 3) exhibited a massive and significant analgesic effect (SMD: −2.35; 95% CI: −4.42 to −0.29). In contrast, the Peripheral Neuropathy subgroup (comprising diabetic neuropathy and carpal tunnel syndrome; k = 6) showed a smaller, non-significant reduction in pain intensity (SMD: −0.38; 95% CI: −0.86 to 0.10). The test for subgroup differences approached significance (*p* = 0.07), suggesting that lesion location (central/spinal vs. peripheral) may be a critical determinant of efficacy.

#### 3.4.2. Subgroup Analysis by Frequency

**Figure 4:**
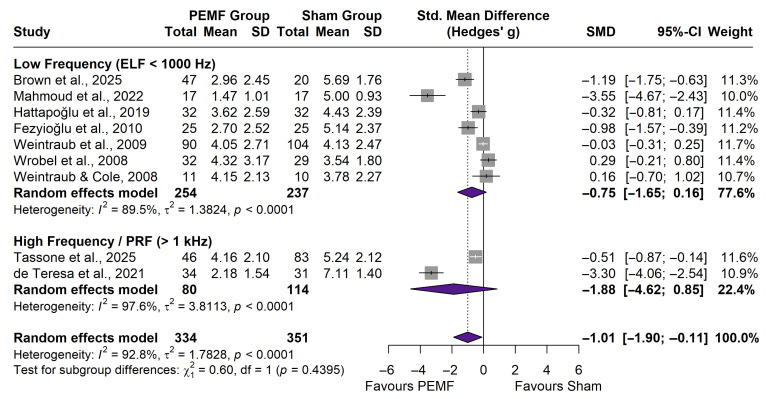
Forest plot of pain intensity by frequency. Subgroup analysis comparing Low-Frequency (ELF) versus High-Frequency/PRF stimulation protocols [10,26,27,29,31,34,35,36,37].

Subgroup analysis based on stimulation parameters (Figure 4) compared Low-Frequency (ELF < 1000 Hz) versus High-Frequency/Radiofrequency (>1 kHz) protocols. Although the High-Frequency subgroup (k = 2) demonstrated a larger point estimate for effect size (SMD: −1.88) compared to Low-Frequency (k = 7; SMD: −0.75), the difference was not statistically significant (*p* = 0.44). Both subgroups exhibited substantial heterogeneity (I^2^ > 89%), preventing definitive conclusions regarding the optimal frequency range.

### 3.5. Secondary Outcomes

**Figure 5:**
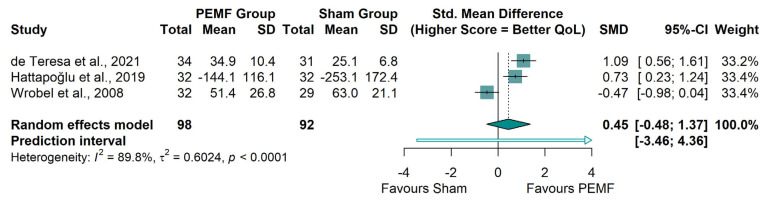
Forest plot of quality of life. Meta-analysis of the effect of PEMF on quality-of-life scores. Scales were standardized such that higher scores indicate better health status [29,35,36].

Quality of Life (QoL): Analysis of functional outcomes (k = 3; *N* = 190) did not demonstrate a significant improvement in QoL for the PEMF group compared to sham (SMD: 0.45; 95% CI: −0.48 to 1.37; *p* = 0.35). Results were highly inconsistent (I^2^ = 89.8%), with only one study showing clear benefits (Figure 5).

### 3.6. Adjuvant Effectiveness and Safety

Due to the lack of reported variance data in 75% of relevant studies, a narrative synthesis was conducted. Four trials consistently reported that the addition of PEMF to standard pharmacological care yielded superior pain relief compared to pharmacotherapy alone, though the magnitude of this add-on effect remains to be quantified.

The intervention demonstrated an excellent safety profile. Detailed safety data for each included trial are summarized in Table 1. Across the nine included studies (*N* = 688), the incidence of device-related adverse events was negligible and identical between groups (0.6% in both PEMF and Sham arms). Only two mild events (transient allodynia) were considered related to the device in the active group [10], while one serious adverse event (myocardial infarction) reported in the Tassone et al. [26] trial was adjudicated as unrelated to the study device. No significant difference in safety endpoints was observed between the experimental and control groups.

It is important to note that while these studies employed a TAU design, implying the continuation of baseline analgesics, the specific protocols regarding medication stabilization or discontinuation were not consistently detailed across all included trials. This lack of granular reporting limits the precision with which the add-on effect can be strictly isolated from potential pharmacological interactions.

### 3.7. Publication Bias and Sensitivity Analysis

**Figure 6:**
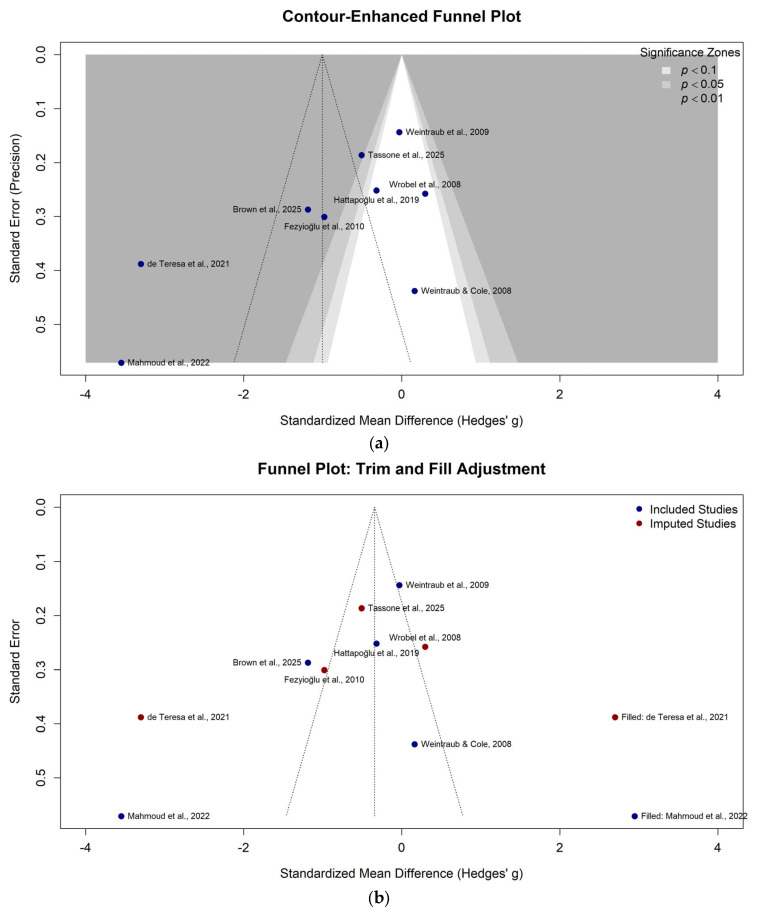
Publication bias assessment. (**a**) Contour-enhanced funnel plot of the included studies. The shaded regions represent significance levels (*p* < 0.1, *p* < 0.05, *p* < 0.01). (**b**) Funnel plot with Trim-and-Fill adjustment, showing observed studies (blue dots) and imputed missing studies (red dots) [10,26,27,29,31,34,35,36,37].

Visual inspection of the contour-enhanced funnel plot (Figure 6a) revealed asymmetry, with a noticeable absence of small studies reporting negative or null results. This was confirmed by Egger’s regression test (*p* < 0.05). To adjust for this potential publication bias, a Trim-and-Fill analysis was performed (Figure 6b), which imputed two theoretical missing studies. Following this adjustment, the overall effect size was attenuated to non-significance (SMD: −0.35; 95% CI: −1.51 to 0.82), suggesting that the primary efficacy results may overestimate the true treatment effect.

### 3.8. Certainty of Evidence (GRADE Assessment)

The certainty of evidence for the primary outcome was evaluated using the GRADE framework. Overall, the quality of evidence was graded as Low for both the Peripheral Neuropathy and Spinal/Radicular Pain subgroups. For the Peripheral Neuropathy subgroup, the certainty was downgraded due to serious risk of bias and serious inconsistency (I^2^ = 80.7%). Similarly, the certainty for the Spinal/Radicular Pain subgroup was assessed as Low, primarily attributed to extreme unexplained heterogeneity (I^2^ = 96.5%) and serious imprecision resulting from a small pooled sample size (*N* = 163). Detailed certainty assessments and downgrade justifications are provided in Supplementary Material File S3.

## 4. Discussion

This meta-analysis demonstrates that the use of PEMF is associated with a statistically significant reduction in pain compared to sham stimulation. However, this overall effect was marked by extreme heterogeneity, and subgroup analyses revealed a clinically important divergence, specifically a massive and significant analgesic effect in patients with spinal and radicular pain versus a smaller and non-significant reduction in those with peripheral neuropathy. From a clinical perspective, the effect size observed in the spinal subgroup (SMD: −2.35) is notable. While statistical significance is crucial, the magnitude of this reduction suggests a benefit that likely exceeds the Minimal Clinically Important Difference (MCID) typically established for chronic pain, implying a tangible improvement in patient functional status beyond mere numerical variation [38].

Safety was excellent, with no serious device-related adverse events. Critically, after adjustment for publication bias, the overall effect of PEMF attenuated to non-significance (SMD: −0.35), suggesting an initial overestimation of the true effect and highlighting the need for caution in interpretation.

The observation of a favorable overall effect of PEMF for neuropathic pain, although attenuated after adjustment for publication bias, is consistent with a developing literature that supports the potential of non-invasive neuromodulation therapies for the management of chronic pain [21]. Previous studies and systematic reviews, despite often having methodological limitations, have suggested that PEMF possesses analgesic properties in various pain conditions including neuropathic pain [11,16]. The capacity of PEMF to modulate neuronal excitability, reduce inflammation, and promote tissue repair could underpin the benefit observed in the global meta-analysis [11,12,15].

### 4.1. Interpreting the Heterogeneity: A Diagnostic Finding

A defining feature of this meta-analysis is the extreme statistical heterogeneity (I^2^ = 92.8%) observed in the global comparison [16,21]. While high heterogeneity is often viewed merely as a statistical limitation stemming from differences in stimulation parameters [9], we interpret this value as a structural diagnostic signal indicating that neuropathic pain is too broad a category to be treated as a monolithic entity in PEMF research. The variance in effect sizes is not attributable to random sampling error but is driven by systematic biological factors.

Our analysis suggests that this heterogeneity is etiologically driven. The statistical noise in the global dataset resolves into a clear, coherent signal when stratified by lesion location. The Spinal/Radicular subgroup exhibited a large and consistent effect size, whereas the Peripheral Neuropathy subgroup consistently showed a non-significant effect. This divergence aligns with the hypothesis that the focal, mechanical nature of spinal radiculopathy may be more amenable to local electromagnetic field modulation than the diffuse, metabolically driven damage seen in diabetic polyneuropathy. Therefore, the high I^2^ should be interpreted clinically as evidence of divergent response patterns: PEMF is not a generic analgesic for all neuropathy, but likely a specific intervention for focal, proximal lesions.

This discrepancy can be explained by the geometric and anatomical mismatch inherent in treating different neuropathic conditions. Spinal and radicular pain typically originate from a focal anatomical target, such as a compressed nerve root, which can be efficiently covered by the magnetic field of a standard solenoid or coil. In stark contrast, peripheral neuropathies, particularly diabetic polyneuropathy, often present in a diffuse and length-dependent stocking and glove distribution. This widespread pathology makes it technically challenging for a localized PEMF device to deliver a therapeutic field intensity across the entire volume of affected nerve fibers, potentially diluting the therapeutic impact in peripheral cohorts [39].

Proposed mechanisms for PEMF action in neuropathic pain are complex and multifactorial, and our findings suggest that these mechanisms might operate differentially according to pain etiology. PEMF has been postulated to influence neuronal excitability, ionic conductance, local blood flow, and the release of neurotransmitters and neuromodulators [11,12,15]. The ability of PEMF to modulate membrane potential and neuronal firing [15] could be particularly relevant in radicular and spinal pain, where nerve compression or local inflammation can generate hyperexcitability. Magnetic stimulation in these regions might help restore neuronal balance and reduce aberrant pain signaling. Moreover, PEMF has demonstrated anti-inflammatory and tissue repair-promoting effects [11].

On the other hand, peripheral neuropathy, especially diabetic neuropathy, often involves more diffuse axonal damage and demyelination [40]. Furthermore, unlike compressive radiculopathies where the lesion is mechanical and focal, diabetic neuropathy is driven by systemic metabolic glucotoxicity and microvascular insufficiency. Localized PEMF applications may successfully modulate neural firing transiently but fail to reverse the ongoing metabolic insult perpetuated by hyperglycemia, potentially limiting its sustained analgesic efficacy in this specific population [41]. It has been suggested that the location of the pain generator within the nervous system is a critical factor in the response to neuromodulatory therapies, with greater efficacy observed when stimulation is applied directly on or near the source of the pain, or in central pain conditions as seen with transcranial magnetic stimulation [42,43].

### 4.2. The Illusion of Efficacy: Publication Bias and Stability

A major concern identified in this analysis is the presence of significant publication bias, confirmed by the asymmetry of the funnel plot and a positive Egger’s regression test (*p* < 0.05). This statistical finding suggests a systemic tendency in the field to publish positive trials while suppressing those with null or negative results (the “file drawer problem”).

The implications of this bias are profound for the interpretation of our results. While our primary unadjusted analysis showed a significant pain reduction, the application of the Trim-and-Fill method, which simulates the inclusion of these missing negative studies, reduced the global effect size to non-significance (Adjusted SMD: −0.35). Clinically, this indicates that the “overall” effectiveness of PEMF reported in the broader literature is likely inflated. The biological effect of PEMF on an unstratified population may be negligible. This instability reinforces our conclusion that relying on global averages is misleading, and clinical utility is likely restricted to the specific, robust responders identified in the spinal subgroup, where the effect size is large enough to withstand potential bias.

### 4.3. Secondary Outcomes and Implementation

Regarding secondary outcomes, the analysis of functional measures revealed a dissociation between pain reduction and the lack of immediate improvement in Quality of Life. This is a well-documented phenomenon in chronic pain research often described as the disability lag. The biopsychosocial model posits that while physiological interventions like PEMF may successfully dampen nociceptive signaling, the restoration of functional domains such as sleep, mood, and social interaction often requires a longer duration of relief and multimodal rehabilitation to manifest. Therefore, the absence of improvement in short-term trials does not necessarily negate the analgesic efficacy of the device but rather reflects the multidimensional complexity of chronic pain syndromes [44].

Finally, regarding implementation, future research must address the cost-effectiveness of PEMF therapy. While traditional high-power systems often require professional administration in clinical settings, the rapid development of portable, user-friendly wearable devices is shifting the paradigm towards home-based self-administration. Validating the efficacy of these home-use systems will be crucial for reducing healthcare costs and improving accessibility for chronic pain patients.

### 4.4. Roadmap for Future Research: Standardization and Design

To advance the field beyond its current fragmented state and address the methodological flaws identified above, future clinical trials must abandon the trial-and-error approach and adopt a rigorous, standardized methodology. Based on the trends identified in this analysis, we propose the following guidelines for future investigation:

1. Parameter standardization (The High-Frequency shift): Researchers should prioritize high-frequency or PRF parameters (>1 kHz) rather than generic ELF stimulation. Our subgroup analysis regarding frequency, while not statistically significant, revealed a notable trend favoring high-frequency and PRF protocols over ELF. This distinction is physiologically plausible because while ELF fields primarily influence local inflammation and microcirculation, high-frequency PRF protocols are known to exert a more direct neuromodulatory effect on synaptic transmission and c-Fos gene expression in the dorsal horn without causing thermal ablation. This suggests that quieting the neural activity in neuropathic pain may require the specific rapid-pulsing characteristics of radiofrequency rather than the slower modulation of ELF [45,46]. Consequently, future trial designs for neuropathic pain should prioritize high-Frequency or PRF parameters (>1 kHz) over ELF protocols, as the current evidence suggests that rapid pulsing is requisite to modulate the pathological synaptic plasticity associated with central sensitization [47].
2. Strict cohort separation: The practice of pooling heterogeneous neuropathic pain patients must end. Future trials should be designed to exclusively recruit either focal/compressive phenotypes (e.g., radiculopathy, mononeuropathy) or diffuse/metabolic phenotypes (e.g., diabetic polyneuropathy). Mixing these groups creates a statistical average that applies to neither patient profile and dilutes the therapeutic signal.
3. Enhanced sham design: Device-based interventions are prone to high placebo response rates due to the sophisticated nature of the treatment ritual. Extensive interaction with medical technology may amplify patient expectations compared to pharmacological trials, contributing to the asymmetry observed in the funnel plot and making the detection of specific therapeutic effects more challenging [48]. Simple devices off shams are no longer sufficient, as patients can often distinguish them due to subtle cues. Future studies should employ sensory-matched active shams, devices that replicate the vibration, sound, or heat of the active unit without delivering the magnetic field, to ensure effective blinding and isolate the true electromagnetic effect from the ritual of therapy.

### 4.5. Limitations

This meta-analysis presents several methodological strengths that reinforce the validity of its findings. It adhered to PRISMA 2020 guidelines for study search, selection, and reporting, ensuring a systematic and transparent review. Comprehensive assessment of bias risk using the Cochrane RoB 2.0 tool allowed for a critical appreciation of the quality of individual studies. Finally, the assessment of evidence certainty using the GRADE framework offers a clear indication of the confidence that can be placed in the results.

Despite its strengths, our meta-analysis has several limitations. Extreme heterogeneity in the primary effect of PEMF versus sham is a major limitation. Variability in intervention protocols among the included studies directly contributes to this inconsistency. Furthermore, the certainty of evidence was rated as low according to GRADE for both subgroups, mainly due to serious risk of bias in some studies, inconsistency, and imprecision. The lack of variance data in 75% of studies relevant to adjuvant efficacy precluded a complete quantitative meta-analysis for this secondary objective, resulting in a narrative synthesis that is inherently less robust [49].

A significant limitation of the current body of evidence is the lack of long-term follow-up data. The RCTs included in this synthesis primarily focused on immediate post-intervention outcomes, with treatment periods ranging from 10 days to 18 weeks. Consequently, there is a lack of evidence regarding the sustainability of the analgesic effect over several months or years. This duration gap represents a critical area for future research, as chronic neuropathic pain requires interventions with demonstrated long-term durability.

Similarly, strict phenotypic characterization remains an unmet need in the field. The majority of included trials relied on global pain intensity scales (e.g., VAS, NRS) without stratifying outcomes by specific neuropathic symptoms. Consequently, it remains unclear whether PEMF is differentially effective against distinct sensory profiles, such as mechanical allodynia, thermal hyperalgesia, or paresthesia, which is information vital for phenotypic-based treatment selection.

## 5. Conclusions

This meta-analysis reveals that, while PEMF therapy demonstrates a statistically significant global reduction in neuropathic pain, this finding must be interpreted with caution due to substantial heterogeneity and identified publication bias. The most clinically relevant discovery is the profound divergence in therapeutic response based on etiology. PEMF exhibited a massive analgesic effect in spinal and radicular pain, likely exceeding the Minimal Clinically Important Difference, whereas its efficacy in peripheral neuropathy was modest and statistically non-significant.

Despite the uncertainty surrounding the overall quality of evidence, the intervention maintains an excellent safety profile, supporting its use as a low-risk adjuvant therapy. However, these results suggest a necessary paradigm shift for future investigation. Future clinical trials must move away from universal low-frequency protocols and prioritize high-Frequency and PRF parameters applied to strictly stratified populations. Specifically, the data support the use of PEMF as a targeted intervention for focal, compressive neural pathologies rather than diffuse metabolic neuropathies. To validate this, future trials must pre-register protocols and publish all outcomes to overcome the systemic bias that currently obscures the true therapeutic scope of this modality.

## Figures and Tables

**Table 1:** Safety profile and adverse events. Incidence and description of adverse events in PEMF versus Sham groups across included studies.

Study	PEMF Group Events (n/N)	Sham Group Events (n/N)	Details of Adverse Events
Brown et al. (2025) [37]	0/50 (0%)	0/21 (0%)	No device-related events.
Tassone et al. (2025) [26]	0/46 (0%)	0/83 (0%)	1 serious event (MI) unrelated to device.
Mahmoud et al. (2022) [31]	0/17 (0%)	0/17 (0%)	None reported.
de Teresa et al. (2021) [36]	0/34 (0%)	0/31 (0%)	None reported.
Hattapoğlu et al. (2019) [35]	0/32 (0%)	0/32 (0%)	None reported.
Fezyioğlu et al. (2010) [34]	0/25 (0%)	0/25 (0%)	None reported.
Weintraub et al. (2009) [10]	2/90 (2.2%)	2/104 (1.9%)	Withdrawal due to allodynia (pain sensitivity).
Wróbel et al. (2008) [29]	0/32 (0%)	0/29 (0%)	None reported.
Weintraub & Cole (2008) [27]	0/11 (0%)	0/10 (0%)	None reported.
TOTAL	2/337 (0.6%)	2/352 (0.6%)	No significant difference.

## Data Availability

The original contributions presented in this study are included in the article/Supplementary Materials. Further inquiries can be directed at the corresponding authors.

## Supplementary Materials

The following supporting information can be downloaded at: https://www.mdpi.com/article/10.3390/neurolint18020028/s1, File S1. PRISMA 2020 Checklist; File S2. Search strategy; File S3. GRADE Evidence Profile.

## Abbreviations

The following abbreviations are used in this manuscript:

CI	Confidence Interval
ELF	Extremely Low-Frequency
GRADE	Grading of Recommendations Assessment, Development and Evaluation
MCID	Minimal Clinically Important Difference
MeSH	Medical Subject Headings
PEMF	Pulsed Electromagnetic Field
PRF	Pulsed Radiofrequency
PRISMA	Preferred Reporting Items for Systematic Reviews and Meta-Analyses
QoL	Quality of Life
RCT	Randomized Controlled Trial
RoB 2	Cochrane Risk of Bias tool (Version 2)
RR	Risk Ratio
SD	Standard Deviation
SMD	Standardized Mean Difference
TAU	Treatment as Usual
