# Supplementary material for: Effectiveness of Pulsed Electromagnetic Field Therapy on Neuropathic Pain: A Systematic Review and Meta-Analysis
Source: Neurol Int. 2026 Feb 6;18(2):28. doi: 10.3390/neurolint18020028 (PMC12943413; doi:10.3390/neurolint18020028)
Supplement: Supplementary file 1 [file neurolint-18-00028-s001.zip › SupplMat_TS3.pdf]

**Table S2.** GRADE Evidence Profile. Certainty of evidence for the effect of PEMF therapy on pain intensity stratified by etiology.

**Question:** Pulsed Electromagnetic Field (PEMF) Therapy compared to Sham Stimulation for Chronic Neuropathic Pain

**Setting:** Clinical Settings

**Bibliography:**

| Certainty assessment | | | | | | | № of patients | | Effect | | Certainty | Importance |
|---|---|---|---|---|---|---|---|---|---|---|---|---|
| № of studies | Study design | Risk of bias | Inconsistency | Indirectness | Imprecision | Other considerations | Pulsed Electromagnetic Field (PEMF) Therapy | Sham Stimulation | Relative (95% CI) | Absolute (95% CI) | | |
| **Peripheral Neuropathy (Diabetic/Carpal)** | | | | | | | | | | | | |
| 6 | randomised trials | serious[a] | serious[b] | not serious | not serious | none | 251 | 271 | - | SMD **0.38 SD lower** (0.86 lower to 0.1 higher) | ⨁⨁◯◯ Low[a,b] | CRITICAL |
| **Spinal/Radicular Pain** | | | | | | | | | | | | |
| 3 | randomised trials | serious[c] | serious[d] | not serious | serious[e] | strong association | 83 | 80 | - | SMD **2.35 SD lower** (4.42 lower to 0.29 lower) | ⨁⨁◯◯ Low[c,d,e] | CRITICAL |

**CI:** confidence interval; **SMD:** standardised mean difference

# Explanations

a. Downgraded one level due to serious limitations in study design. While some trials had low risk of bias, Tassone et al. (2025 )[1] and Fezyioğlu et al. (2010) [2] exhibited high overall risk. Specifically, Tassone et al. presented high risk regarding deviations from the intended intervention (Domain 2) and selection of the reported result (Domain 5), while Fezyioğlu et al. showed high risk in the randomization process (Domain 1), introducing potential bias to the pooled estimate.

b. Downgraded one level due to unexplained heterogeneity (I^2 = 80.7%). The included trials report conflicting conclusions: Brown et al. (2025) [3] and Fezyioğlu et al. (2010) [2] reported significant therapeutic effects, whereas larger trials such as Weintraub et al. (2009) [4] and Wróbel et al. (2008) [5] found no significant difference between groups, suggesting variability in response across peripheral neuropathies.

c. Downgraded one level due to study limitations. Two of the three included studies (Mahmoud et al., 2022 [6]; Hattapoğlu et al., 2019 [7]) were assessed as having "some concerns" in bias domains related to deviations from intended interventions and the randomization process, which affects the reliability of the aggregated results.

d. Downgraded one level due to extreme heterogeneity (I^2 = 96.5%). The magnitude of the effect varied substantially across studies: Mahmoud et al. (2022) [6] and de Teresa et al. (2021) [8] reported very large effect sizes (SMD < -3.0), contrasting sharply with Hattapoğlu et al. (2019) [7], who found a negligible and non-significant effect.

e. Downgraded one level due to serious imprecision. The total pooled sample size ($n$ = 163) is relatively small (below the optimal information size of 400 participants), leading to a wide confidence interval that reduces certainty in the precise magnitude of the effect.

## References


1. Tassone, E.E.; Page, J.C.; Slepian, M.J. Assessing the effects of pulsed electromagnetic therapy on painful diabetic distal symmetric peripheral neuropathy: A double-blind randomized controlled trial. *J. Diabetes Sci. Technol.* **2025**, *19*, 361–369.
2. Fezyioğlu, P.; Özdemir, F.; Güldiken, S.; Balcı, K.; Süt, N. The effects of pulsed electromagnetic field treatment in pain due to diabetic polyneuropathy. *Trakya Univ. Tip Fak. Derg.* **2010**, *27*, 227–233.
3. Brown, L.; Gage, E.; Cordner, H.; Kapural, L.; Rosenberg, J.; Bedder, M. Safety and efficacy of magnetic peripheral nerve stimulation for treating painful diabetic neuropathy. *Neuromodulation* **2025**, *28*, 1366–1373. https://doi.org/10.1016/j.neurom.2025.03.074.
4. Weintraub, M.I.; Herrmann, D.N.; Smith, A.G.; Backonja, M.M.; Cole, S.P. Pulsed electromagnetic fields to reduce diabetic neuropathic pain and stimulate neuronal repair: A randomized controlled trial. *Arch. Phys. Med. Rehabil.* **2009**, *90*, 1102–1109.
5. Wróbel, M.P.; Szymborska-Kajanek, A.; Wystrychowski, G.; Biniszkiewicz, T.; Sieroń-Stołtny, K.; Sieroń, A.; Pierzchała, K.; Grzeszczak, W.; Strojek, K. Impact of low frequency pulsed magnetic fields on pain intensity, quality of life and sleep disturbances in patients with painful diabetic polyneuropathy. *Diabetes Metab.* **2008**, *34*, 349–354.
6. Mahmoud, L.S.E.D.; Habib, K.A.; Nagy, M.R.; Badawy, M.S. Effect of electromagnetic field therapy on neck pain and proprioception in cervical radiculopathy patients: A randomized controlled trial. *NeuroQuantology* **2022**, *20*, 824–831.
7. Hattapoğlu, E.; Batmaz, İ.; Dilek, B.; Karakoç, M.; Em, S.; Çevik, R. Efficiency of pulsed electromagnetic fields on pain, disability, anxiety, depression, and quality of life in patients with cervical disc herniation: A randomized controlled study. *Turk. J. Med. Sci.* **2019**, *49*, 1095–1101.
8. de Teresa, C.; Varela-López, A.; Rios-Álvarez, S.; Gálvez, R.; Maire, C.; Gracia-Villar, S.; Battino, M.; Quiles, J.L. Evaluation of the analgesic efficacy of a bioelectronic device in non-specific chronic low back pain with neuropathic component. A randomized trial. *J. Clin. Med.* **2021**, *10*, 1781.
